# Supplementary material for: Umbilical cord characteristics and their association with adverse pregnancy outcomes: A systematic review and meta-analysis
Source: PLoS One. 2020 Sep 24;15(9):e0239630. doi: 10.1371/journal.pone.0239630 (PMC7514048; doi:10.1371/journal.pone.0239630)
Supplement: S1 Appendix — (DOCX) [file pone.0239630.s002.docx]

**Appendix A - example Medline search for cord abnormalities and adverse outcomes**

1. Umbilical cord; 40300 results
2. 2 AND (loop* OR knot* OR nuchal OR short OR long OR coil* OR hypercoil* OR hypocoil* OR tangle* OR entangle* OR twist* OR thick* OR lean OR morpholog* OR diameter OR large OR insert*); 7956 results
3. Umbilical cord/anatomy and histology; 9915 results
4. 2 OR 3; 15143 results
5. 4 AND (small for gestational age OR SGA OR IUGR OR intrauterine growth restriction OR fetal growth restriction OR FGR OR small for date* OR SFD OR low birth weight OR LBW OR VLBW OR stillbirth); 1300 results
6. 5 AND (pregnancy complications/ OR pregnancy outcome/); 976 results
7. 4 AND infant, low birth weight/ OR infant, small for gestational age/ OR stillbirth/; 251 results
8. 6 OR 7; 1127 results
9. 8 NOT (stem adj cell* OR zika OR zika adj virus OR huvec OR huvec cell* OR twin.ti,ab OR twin adj pregnanc*.ti,ab);796 results
10. 10 OR “umbilical cord pathology and stillbirth”; 858 results
