# Supplementary material for: Umbilical cord characteristics and their association with adverse pregnancy outcomes: A systematic review and meta-analysis
Source: PLoS One. 2020 Sep 24;15(9):e0239630. doi: 10.1371/journal.pone.0239630 (PMC7514048; doi:10.1371/journal.pone.0239630)
Supplement: S2 Data — (DOCX) [file pone.0239630.s004.docx]

**QUADAS-2 for studies of ultrasound accuracy for detection of nuchal cord**

| **Study** | **Risk of bias** | | | | **Applicability concerns** | | |
| --- | --- | --- | --- | --- | --- | --- | --- |
|  | **Patient selection** | **Index test** | **Reference standard** | **Flow and timing** | **Patient selection** | **Index test** | **Reference standard** |
| Abdallah 2018 | Low | Unclear | Unclear | Low | Low | Low | Low |
| Akkaya 2017 | High | High | Unclear | Low | Low | Low | Low |
| Aksoy 2003 | Low | Unclear | Unclear | Low | Unclear | Low | Low |
| Assimakopoulos 2005 | Low | Low | Low | Low | Low | Low | Unclear |
| Clapp 2003 | Low | Low | Low | Low | Low | Low | Unclear |
| Gonzalez-Quintero 2004 | High | Unclear | Unclear | Unclear | Low | Low | Unclear |
| Hanaoka 2002 | Low | Unclear | Unclear | Low | Low | Low | Unclear |
| Lal 2007 | Low | Low | Unclear | Low | Low | Low | Unclear |
| Markov 2007 | Unclear | Unclear | Low | Unclear | Unclear | Unclear | Unclear |
| Peregrine 2005 | Unclear | Low | Unclear | Low | Unclear | Low | Low |
| Qin 2000 | Low | Low | Low | Low | Low | Low | Low |
| Romero Gutierrez 2000 | Low | Low | Unclear | Unclear | Unclear | Unclear | Unclear |
