# Supplementary material for: Umbilical cord characteristics and their association with adverse pregnancy outcomes: A systematic review and meta-analysis
Source: PLoS One. 2020 Sep 24;15(9):e0239630. doi: 10.1371/journal.pone.0239630 (PMC7514048; doi:10.1371/journal.pone.0239630)
Supplement: Attachment — Submitted filename: Response to Reviewers.docx [file pone.0239630.s005.docx]

**PLOS ONE Review of Umbilical Cord Characteristics and Their Association with Adverse Pregnancy Outcomes: A Systematic Review and Meta-Analysis**

Reviewer #1:

1. The heterogeneity of the studies included (see lines #143-148) (and graded subjectively by the authors as poor (n=7), fair (n=103), and good (n=35), in my assessment negates any statistical analysis in aggregate (essentially, the gist of this submission). I note the authors themselves acknowledge this major drawback (see line # 162 "Heterogeneity was very high").

Respectfully, we do not agree that statistical analysis is negated by high heterogeneity. Line 162 (now line 181 in the manuscript with tracked changes) refers to the heterogeneity from the analysis for nuchal cord incidence only, although many of our analyses also showed very high heterogeneity; the estimate from this analysis is consistent with the literature, suggesting that this heterogeneity has not significantly affected the estimate. The heterogeneity itself is also an important finding as it may demonstrate a lack of high quality studies, which current guidance is built upon, and may signify that confounding factors are responsible for some of the variation seen in effect sizes.

The grades of poor, fair, or good refer to the NIH quality assessment tools specified in the methods; this has been clarified (line 159-160).

2. The primary outcome criteria assessed "stillbirth" (see line # ), clearly falls short of the outcome as suggested in the title "adverse perinatal outcome". The authors later in the manuscript address "fetal compromise" (see lines # 351-361). Tools applied in these analyses are incomplete (see next point). The statement "and the observed effects sizes were comparatively modest" is unsubstantiated and lacks any clinical significance.

The ‘adverse perinatal outcome’ in the title was chosen to indicate that in this review we looked at several adverse outcomes, of which stillbirth is the most severe. We think that stillbirth is a most useful marker of adverse pregnancy outcome, since most observers can agree on its definition, at least within a given range of gestations.

We also collected data on several secondary outcomes, although data for all outcomes were not collected by all studies. This is also an important finding and emphasises the need for a core outcome set in studies of this nature.

The first mention of modest effect sizes has been removed (line 395) and this is discussed later on in the context of the triple risk model (ref. 60) where risk factors with odds ratios between 2 and 3 are unlikely to be definitive causes of stillbirth.

3. Apgar scores are highly subjective. More precise and scientifically correct perinatal outcome include umbilical artery pH and base excess, both absent in the data assessed in this submission.

We agree that Apgar scores are subjective; however, they are almost universally used clinically and are widely reported in studies. In addition, there is an established relationship between Apgar scores and the presence of metabolic acidosis (Cnattingius et al. J Perinat Med 2020 Jun 25;48(5):514-515), as well as neonatal outcome (Razaz et al. BMJ 2019 May 7;365:l1656 and Persson et al. BMJ. 2018 Feb 7;360:k207) Clearly, if we were prospectively designing studies of pregnancy outcome with umbilical cord problems, we would include measurements of umbilical artery pH and base excess in order to best correlate the observed Apgar scores to fetal hypoxia or asphyxia.

4. It is methodologically incorrect to include cord prolapse in the context of "umbilical cord abnormalities" (see lines # 180-181 and later lines # 338-340), (often associated with artificial rupture of the membranes - ie, iatrogenic) in the analysis of nuchal and true knots of the umbilical cord (both spontaneous events).

We included cord prolapse in this review as, pathologically, it may still affect fetuses in a similar way to other cord abnormalities, whether spontaneous or not. We do not limit our study to abnormalities intrinsic to the cord; in fact, the tightening of a true knot of the cord, perhaps during birth, is not all that dissimilar to a prolapsed cord in, say, a footling breech presentation. We agree that if rupture of membranes with a floating presenting part causes cord prolapse then this is iatrogenic, yet the cord position prior to this rupture is analogous to the cord position in which birth causes a nuchal cord to tighten.

We only found enough data to report on the incidence of cord prolapse and, as such, we could not relate it to adverse outcome.

5. Data pertaining to umbilical cord length are incomplete.

6. Data pertaining to prenatal sonography of nuchal cords and true knots of the umbilical cord, are incomplete.

We are unsure what ‘incomplete’ means in the context of these comments. Umbilical cord length was not recorded by every study; nevertheless, we have reported all the available data from our included papers. Similarly, only four studies presented usable data to analyse the ultrasound accuracy of detecting true knots.

7. The statement in line # regarding the relative higher sensitivity rates associated with intrapartum prenatal sonographic diagnosis (lines # 220-222) reflect the shorter time from diagnosis to delivery. Worded differently, a nuchal cord throughout gestation is a dynamic condition, often not seen at repeat sonographic assessment (and conversely).

We agree that higher sensitivity is likely to be due to this shorter interval; however, we were not able to test this explicitly. This was stated in the discussion.

8. I agree with the authors that tightness of the umbilical cord is highly subjective and cannot be addressed (lines # 246 -248).

We agree that this review is limited by study definitions of nuchal cord tightness and subjective measures such as whether the cord could be slipped over the fetal head; more useful measures such as classifying nuchal cords as type A or B are necessary.

9. The increased likelihood of stillbirth in cases of true knot of the umbilical cord (lines # 282-285), is well-established.

This association has been seen in cohort studies but has not been confirmed in a meta-analysis across different study settings until now. We would argue that meta-analysis provides a more reliable measure than single studies and therefore adds to the existing evidence.

10. Lines # 363-369): Respectfully, I differ with the authors, in that this "systematic review" is weak (the fact that a pre-specified protocol by an international multidisciplinary review team, is irrelevant). A weak paper cannot be strengthened by these measures. In contrast, I do agree with the authors regarding the notable limitations of this submission - namely the heterogeneity of the included studies reflected in the variations of definitions utilized and associated outcomes.

We agree that any systematic review is limited by the quality of its included studies, and in this review most are of fair quality. The problem, as the reviewer states, lies with these varying definitions and this is something that needs to be addressed by future studies – which hopefully this review can help bring to researchers’ attention.

However, we do not agree that a pre-specified protocol (meaning that analyses and outcomes cannot be changed post-hoc in the name of finding significant results) and an international multidisciplinary team are irrelevant. Our team’s expertise was vital in helping to appraise a wide range of included studies and to interpret our results.

11. Similarly, as the authors correctly state (lines # 368-369), all data presented may reflect publication bias.

We agree and feel like this is an important use of systematic reviews, to bring attention to areas in the literature which may be affected by publication bias.

12. Clinical implications stated by the authors (lines # 389-413), are well recognized within the Obstetrical community and do not reflect original previously unpublished data.

These associations have not been shown in a meta-analysis until now, especially the effect of nuchal cord and number of nuchal loops.

13. Line # 84: "Death of a baby" should be simply "stillbirth", or "intrauterine fetal demise".

This line is our study definition for stillbirth or intrauterine fetal death, and as such we prefer to leave it as is.

14. Results: (line # 28): A sentence should not begin with numbers.

This has been changed to ‘This review included 145 studies.’

15. The final sentence of the Results section of the Abstract (lines # 34-35) is incomplete as written.

We have made modifications to the abstract based on this and other reviewers’ comments; in order to make these edits and stay within the word limit, this line has been removed.

16. Abstract: The final comment regarding the need for future studies (lines 39-40), merely reflects the weakness of the secondary outcomes assessed.

This was touched on in our response to #3 but we note that the lack of data for certain outcomes is a function of the available literature rather than our outcome set. We have changed this sentence to clarify that we have identified areas for future research, rather than failed to find data due to our specified outcomes (line 40-41).

17. The first sentence of the Conclusion (line # 37) is incorrect as written. Pregnancy itself "is associated with stillbirth". It appears that the authors intend to state: "True umbilical cord knots are associated with an increased risk of stillbirth". Regarding the remainder of the sentence (lines # 37-38) "the incidence of stillbirth is higher with multiple nuchal loops". Higher than what? Please clarify. It appears the authors mean to state, "in contrast to cases of a single nuchal cord, multiple nuchal loops have an increased risk of stillbirth".

Thank you for identifying this error; this sentence has now been clarified (line 38-39).

18. The tables are exhaustive, and in my assessment do not contribute significantly to this effort. For example. regarding Lolis et al (1998), why would umbilical cord length increase with parity. I question whether such a "finding" is worthy of quoting?

The characteristics of included studies table (Table 1) needs to contain every relevant study, and although it does take up a lot of space this is a requirement of a systematic review. In response to the question, cord length may increase with parity as multiple studies have correlated cord length with fetal size, which is known to increase with parity. Hence, parity may be an important confounder in studies of cord length; however, the included studies did not provide enough data to look at this effect.

**Reviewer #2:** The authors of the manuscript entitled “Umbilical cord characteristics and their association with adverse pregnancy outcomes: a Systematic review and meta-analysis” have produced a fine analysis of literature published in the last 50 years on the impact of umbilical cord abnormalities on stillbirths and other related adverse pregnancy outcomes.

Thank you for your positive views regarding our manuscript.

There are a few comments: 1. Robert W Bendon is lacking his institutional address.

Dr. Bendon is retired; this has now been clarified/added.

2. Introduction: page 4, line 63: duration of UCA: this is most difficult to know/assess (unless cord prolapse). I suggest to delete this element as a feature of variation in published results

We agree that this is very difficult to know; however, it may still be a cause of variation. We feel that it should remain reported as an “unknown confounder” as it could be as important as those which are known.

3. M&M: page 5, line 87: low birth weight “at term “(<2500g)
4. Table 1: reference number to each paper included should be added
5. Tables 2: clarify at the bottom of the table: NC: nuchal cords
6. Table 3: clarify at the bottom of the Table the meaning of: CS, PTB, NICU, BW, SGA
7. Table 4: clarify at the bottom of the Table the meaning of: PTB, NICU, LBW, SGA/FGR, OR

These have all been added; reference numbers are next to the authors’ names in Table 1 and keys have been put beneath tables 2-4.

8. Although Tables 2, 3 and 4 include the significant findings in bold, the readers would benefit from the addition of a Table with the findings related to UCA, which would be better placed at the end of SYNTHESIS OF RESULTS. I.E:- Loose nuchal loops were more frequent than tight loops, with a summary frequency of 10% (95% CI 4, 18) compared to 5% (95% CI 4, 77). - Cord prolapse incidence was 0.17% - True knots at birth incidence was 1% - etc.

Thank you for the suggestion. We have added a summary of these results as Table 5, although we have only summarised UCA and stillbirth data along with incidences and normal characteristics so as not to repeat data from the other tables.

**Reviewer 4**

**Overview**

The authors’ objective was to investigate the association between umbilical cord abnormalities and adverse pregnancy outcomes, including stillbirth, cesarean section, 1-minute Apgar score under 7, 5-minute Apgar score under 7, and NICU admissions. To achieve this objective, a systematic review/meta-analysis was performed. The main results were the presence of nuchal cords in 22% of births, no evidence of an association between stillbirth and any nuchal cord, and higher odds of stillbirth in pregnancies with a true cord knot. Other results included the associations of true knots and preterm birth, 5 minute Apgar score under 7, NICU admissions, low birth weight, and small for gestational age, multiple or tight nuchal loops with 1 minute Apgar score under 7, and abnormally coiled cords with preterm birth, low Apgar scores, low birth weight, small for gestational age, and cesarean section.

The authors should be commended, as they investigated the relationship between multiple exposures and outcomes. This literature synthesis was a huge undertaking. This research would be of interest to the audience of PLOS ONE given the unique topic and rigorous methodology. It summarizes the current research on umbilical cord abnormalities and adverse pregnancy/birth outcomes while also providing a, potentially more accurate and complete, pooled estimate of the associations. However, the poorly written, incredible long results section lessens the quality of the paper and requires major reworking and edits to allow readers to identify key, clinically important results.

Thank you for your comments and thorough review.

**Abstract**

Please provide the following edits:

- In the objective, please mention this is also a meta-analysis not just a systematic review.
- In the results, the authors mention that true knots, multiple, or tight nuchal loops and abnormally coiled cords were associated with secondary outcomes. However, what the secondary outcomes are is not mentioned in the abstract. Secondary outcomes should be more clearly listed in the methods. Alternatively, secondary outcomes and/or significant associations with cord abnormalities could be listed in the results section.
- Case control studies were also eligible for inclusion (not just cohort or cross-sectional studies). This should be stated more clearly in the abstract.
- In the final sentence, the word “outcome” should be plural.

These changes have now been made to the abstract, secondary outcomes are listed in the methods section (lines 87-91), and this line has been removed from the abstract in order to incorporate all reviewers’ comments while staying within the word limit.

**Introduction**

The introduction was well written and provided an excellent overview of umbilical cord abnormalities and the conflicting research on their association with adverse pregnancy outcomes. The authors clearly demonstrate the need for study and justify the investigated exposures and outcomes. Minor comments and suggestions that could strengthen the effectiveness of the introduction and further outline the literature gaps and rationale for the study are below.

- When discussing true knots and cord prolapses’ link to adverse outcomes, a short statement on the outcomes they are linked to would be beneficial.
- Additional information on the potential biological mechanisms linking these abnormalities to stillbirth and other adverse outcomes, either in the introduction or the discussion would provide further support for the investigation.

A statement on the links to adverse outcomes has been added in lines 56-58. Potential biological mechanisms are mentioned in the clinical implications section of the discussion and in the outcomes section of the methods as justification for their selection (lines 111-115).

**Methods**

The authors performed a systematic review and meta-analysis of the relationship between umbilical cord abnormalities and adverse pregnancy outcomes. Exposures were all nuchal loops, single loops, multiple loops, tight or loose loops, true knots, hypocoiling, and hypercoiling. The primary outcome was stillbirth (or intrauterine fetal death) and secondary outcomes included neonatal intensive care unit admission, preterm birth, small for gestational age, low birth weight, low Apgar score (<7 at 1 minute and 5 minutes), and frequency of cesarean birth. MEDLINE, EMBASE, CINAHL, and Google Scholar were searched from 1960 to December 1^st^, 2019. Reference lists of included studies and grey literature was also searched. Cohort, case control, and cross-sectional studies of singleton pregnancies 20 weeks gestation or older that reported on the frequency of umbilical cord characteristics or cord abnormalities and their relationship to adverse outcomes were included. NIH quality assessments tools and QUADAS-2 were used to assess the quality of included studies. Statistical analyses, including random effects meta-analyses, I^2^ calculation, and calculation of incidence rates, were performed in STATA. Questions, comments, and minor suggestions are listed below.

- The methods section would be more effective and clearer if the paragraphs were reordered and additional headers were added. Specifically, the second paragraph, discussing primary and secondary outcomes, should either go before the “eligibility criteria, information sources, search strategy” header under a new header (e.g. “Outcomes”) or after the paragraph describing the search strategy.

Thank you for this suggestion; this has been moved to a section titled ‘outcomes of interest’ after the search strategy (line 105 onwards).

- The multiple exposures (i.e. nuchal cords, single loop, multiple loops, true knots, tight or loose loops, hypo- and hypercoiling) should be stated in methods prior to the outcomes.

We have added a line (85-85) to clarify that all other UCA were eligible for inclusion, in order to be consistent with our protocol (where it is detailed that variations such as number of nuchal loops will also be recorded).

- To allow readers to better gauge the quality of the search and manuscript, the exact searches, including the use of any MESH terms, for each database (i.e. MEDLINE, EMBASE, CINAHL, and Google Scholar) need to be provided in the appendix.

We do not have access to CINAHL currently but have expanded the appendix to include the Embase search strategy and a list of keywords used to search Google Scholar. The Medline search was the most detailed due to the nature of the database and was used to inform the searches in other databases.

- The authors mention that studies not published in English were translated where possible. How many studies were not in English? How many could not be translated?

We did not include any papers where translation was required so this was not detailed in our PRISMA diagram. No papers were excluded because they could not be translated.

- The authors should specify that both the NIH quality assessment tool for cohort and cross-sectional studies and the NIH quality assessment tool for case-control studies were used (i.e. two separate tools).

This has been clarified (lines 127-128).

- A description of the scores/cutoffs used to classify studies as good, fair, or poor for NIH quality assessment is necessary.

The guidance for the NIH quality assessment tools state that arbitrary cutoffs should not be used; instead, the risk of bias potential for each domain should be considered as well as any ‘fatal flaws’. Due to this, we do not feel as though this can be included as the NIH tool allows reviewers to make decisions without being limited by a scoring system; however, some of the areas where studies were most likely to be marked down are described in the results section (lines 160-163).

- The statement regarding what an I^2^ value describes is misleading. The I^2^ value describes the amount of total variability in (risk) estimates that is due to heterogeneity between studies rather than by chance. This heterogeneity could be due to many factors, not just differences in the true exposure effect.

This has been amended to “I^2^, derived from Cochran’s chi-squared statistic Q, was calculated to describe the percentage of variability in effect estimates that is due to heterogeneity”.

- Since the authors anticipated differences in definitions of stillbirth/intrauterine fetal death, potentially by geographic location, the reviewer suggests sensitivity analyses separating studies by either geographical location of the study or the definition of stillbirth/intrauterine fetal death (if numbers permit).

Thank you for the suggestion; however, after evaluating the number of studies we have for most analyses, we do not deem that these sub-group analyses would be possible.

**Results**

The key results included 1-nuchal cord presence in 22% of births, 2-no evidence of an association between any nuchal cords and stillbirth, 3-a statistically significant association between stillbirth and true cord knots, and 4-the diagnostic accuracy of antenatal or antepartum ultrasound for the detection of cord abnormalities was modest, though the diagnosis of nuchal cord was most accurate when performed in early labor. The major issue with this paper is the presentation and highlighting of important results. This section is poorly written, detracting from the important results it contains. Briefly, there are many run-on sentences that should be split into two separate sentences, separated with a semicolon, or split with a comma and a coordinating conjunction. Several sentences started with numbers, which should be spelled out. There are a few sections describing umbilical abnormalities for which an incidence or association between abnormalities and outcomes could not be calculated. This should be stated in a more clear, concise manner in each section and/or could be stated once with all associations for which meta-analyses could not be performed listed together instead of in separate sections. Abbreviations should be defined at the first use (e.g. UCI). There are also errors in citation brackets (e.g. splitting references like on page 16). The results section was extremely dense, making it difficult to clearly identify important results. The reviewer suggests a rewrite of the section to increase clarity and conciseness and enable readers to quickly identify the critical, clinically relevant results. Suggestions, questions, and comments which need to be addressed are below.

This section has been heavily edited to make it more concise, and an introductory sentence (lines 165-167) has been added to the synthesis of results section to enable readers to more easily find the results of interest. Unnecessary information that is otherwise presented in Table 1 has been removed (see the nuchal cord section line 178 onwards for an example). In sections where analysis was not possible, this is now stated right away (for example abnormal length, line 210 onwards), allowing readers to move through this section more easily.

- There is a lack of information on why over 2,000 studies were excluded based on title and abstract. More information is necessary (e.g. how many did not investigate umbilical cord abnormalities or the primary or secondary outcomes? How many were not in English? How many were excluded because the study population contained twins? How many were not observational study designs (e.g. reviews, other meta-analyses, case series, etc.)?).

The reasons for exclusion were not recorded at the title/abstract stage as this is not convention and would make the initial screening process far too time-consuming when working with such numbers of references; however, these reasons were recorded at the full text screening stage and can be seen in Figure 1.

- For articles excluded after full text review, what were the reasons for the authors inability to extract data from 11 studies (was data simply not present or in a usable format?)? Nineteen studies were excluded for other reasons, what were the other reasons?

“Data could not be extracted” denotes that data were not in a usable format. Figure 1 has been modified and the ‘other’ section has been combined with the ‘not relevant’ section to remove any confusion. This was an inconsistency in the terms used between multiple reviewers.

- In Figure 1, since the authors had multiple outcomes, the “adverse outcomes and UCA (n=90)” statement should be further broken down into the numbers used for each outcome (e.g. stillbirth, low birth weight, etc.)

We feel as though the overlap between studies and the introduction of more numbers would be too confusing to readers seeing as this section is already broken down into subcategories.

- Please address the following: what were the different gestational ages used in the average cord length calculation? Were there numerous studies that focused on preterm pregnancies/births included in this calculation? There is some evidence that length is correlated with gestational age (Georgiadis et al. 2014). If numbers permit, it would be interesting to look at differences in average cord length based on gestational age (or term vs preterm).

There was a wide range of gestational ages used in this calculation, which has been clarified, and studies also used different classifications (e.g. some were at ‘term’, some were an average gestation, some were all births after 28 weeks). This uncertainty was partially why we also analysed all studies where cord was measured at 39 weeks, to see if this estimate was significantly different.

It is likely that some preterm births were included due to the definitions mentioned; however, these papers did not present these data separately. We agree that this would be an interesting topic for a future study.

The abnormal coiling section should be rewritten, as the current sentence structure does not clearly state that a pooled incidence was not calculated due to differences in definitions of hypo- and hypercoiling.

This section has been rewritten accordingly.

- Twelve studies were included in the diagnostic accuracy of ultrasound for nuchal cords, with ultrasound screening occurring at any gestation. What was the range in gestational ages at which ultrasound screening took place?

Again, this varied; however, in all but two studies, measurement was after 36 weeks. This has been added (lines 254-255).

- For diagnostic accuracy of ultrasounds for nuchal cords and true knots, was author contact undertaken when requisite data was not provided or where they simply excluded from analysis on diagnostic accuracy?

Where data were not provided in a usable form for diagnostic accuracy analysis, these papers were excluded from the study. This was not a planned analysis, and was added once data were obtained/extracted and analysis deemed possible.

- The I^2^ for random effects models for the primary and secondary outcomes analyses should be reported, along with an interpretation of their meaning.

Descriptions of heterogeneity values have been added to sections where they were missing (lines 205 and 298 for example) and I-squared values have been added to tables 2 and 4, which have been reformatted.

- Was the analysis looking at multiple versus no loops and a single loop planned a priori or completed ad hoc after finding no association between one loop and stillbirth?

This analysis was planned and can be seen in the subgroup analysis of the review protocol <https://www.crd.york.ac.uk/prospero/display_record.php?RecordID=99049>

- For single, multiple, tight, and loose loops association with the secondary outcomes, odds ratios, confidence intervals, I^2^ values, and p values should be included in the text instead of simply stating their association or lack therefore (paragraph 2 on page 13).

I-squared and p values have been added to the nuchal cord and stillbirth paragraph as this is our primary outcome (line 263 onwards). Data for other outcomes are mainly in Table 2 to make this section shorter, as suggested.

- The final sentence in the third paragraph on page 13 is confusing. There are three estimates of heterogeneity. What association is each measure for?

This paragraph has been rewritten to clarify the meaning (line 298-300).

- Only a single odds ratio and confidence interval is given following the statement regarding fetal growth restriction and small for gestational age associations with nuchal cords. Were the nine studies that measured these outcomes combined into a single analysis or were fetal growth restriction and small for gestational age estimates pooled in two separate analyses?

The studies that measured SGA/FGR were combined as they used a range of definitions; this has been clarified in line 309.

- In the abnormal coiling and other adverse outcomes section, do the authors mean >90^th^ centile when discussing thresholds of hypo- and hypercoiling?

There was a range of definitions used by the different studies; however we combined them as variation was minimal (please see lines 344-349).

- The multiple headings stating the specific abnormality and adverse outcomes were helpful. To further increase clarity and shorten the results section, please consider the following:
- Reference tables and include citations as opposed to continually listing the number of studies and pregnancies in each analysis.
- Highlight significantly, clinically meaningful associations and only include short statements on nonsignificant associations and associations which could not be gauged due to insufficient data.
- Justification for exclusion of specific studies analyses could be moved to an appendix or listed in the methods.
- List odds ratios, 95% confidence intervals, I^2^, and p values for all significant associations.

Numbers of studies/pregnancies have been removed where appropriate (e.g. line 304, line 318) and I-squared and p values added (see various comments above).

**Discussion**

The discussion provides a nice summary of the major results of the meta-analysis. To improve the quality and interpretation of the study results, please address the following:

- In the first paragraph which summarizes the results, please consider adding all major, significant (risk) associations. As there are numerous significant associations, the authors could limit those discussed in the discussion to the highest odds ratios, those low I^2^ values, and/or those with the most clinical importance.

We feel as though this would add unnecessary length and repetition to the manuscript; however, we have added a summary results table at the end of the results section (table 5).

- The discussion of the strengths and limitations of the research was thorough and well done. Depending on the included studies, the wide variety of locations and population demographics may also be a strength.

Thank you for your comment. We agree that it is good to have data from a range of locations. Unfortunately, we are unable to perform a formal subgroup analysis on geographic location or demographics as suggested by other reviewers as there were insufficient studies to allow this.

- In the introduction, the authors state that some classification systems for stillbirth do not include umbilical cord abnormalities as a potential cause of death. A discussion how estimates could be biased by differences in classification systems for stillbirth would strengthen the discussion.

We have added a section to the discussion, where we explain that estimates of the contribution of umbilical cord problems to stillbirth are made more complex by differences in the classification systems for stillbirth, some of which do not include umbilical cord complications. We have added a reference to a recent paper from the Stillbirth Collaborative Research Network which identifies that 19% of stillbirths had cord complications in their study.

- In the clinical implications section, a brief reiteration on how the investigated abnormalities, prior to discussion of the triple risk model, could lead to adverse outcomes would be advantageous. Figure 2 could also be referenced in this section.

We have ensured that the clinical implications section describes how the investigated abnormalities could lead to adverse outcomes. We have referenced Figure 2 in this section.

**Conclusion**

This section provides a flawless end to the paper and is very well written. Thank you.

**Tables, Figures, and Supporting Information**

- In Table 1, location of the study and/or ancestry, race, or ethnicity of the participants, statistical methods (including covariates adjusted for), and major results/effect estimates would be beneficial.

As mentioned, we have looked into study location; however, subgroup analysis is not possible due to the small number of studies. We have also received comments from other reviewers that the tables are rather extensive as they are, and therefore, do not wish to expand them.

- Tables 2, 3, and 4 all contain estimates of umbilical cord abnormalities and the risk of adverse neonatal outcomes; however, they are all in different formats. A consistent format should be used for all tables.
- Table 2 and 4 need to include I^2^ and p values (format similar to Table 3).
- All tables should have footnotes with additional information, including abbreviations and descriptions of what the shading and bold text represent.

Tables 2 and 4 have been reformatted.

- Appendix A should contain all exact searches for each association using each search engine/database.

This has been addressed in an earlier comment.

- The all data file is well organized and very thorough and provides all the information necessary to enable other researchers to recreate the analyses.

**Miscellaneous**

- The author Robert W Bendon needs an affiliation

This has been added as ‘Retired from Norton Children’s Hospital, Louisville, USA’

- Major edit: the paper needs to cite all 145 studies included in the various meta-analyses.

Citations for all papers have been added to Table 1.
